# Supplementary material for: Methods for processing and analyzing images of vascularized micro-organ and tumor systems
Source: Front Bioeng Biotechnol. 2025 Jun 12;13:1585003. doi: 10.3389/fbioe.2025.1585003 (PMC12198201; doi:10.3389/fbioe.2025.1585003)
Supplement: Supplementary file 1 [file DataSheet1.pdf]

Supplementary Material

1 Installing the HughesLabTools from GitHub

Download Folder from GitHub

1. Go to https://github.com/shachey13/HughesLabTools
2. Go to the Green Code Button and click the down arrow

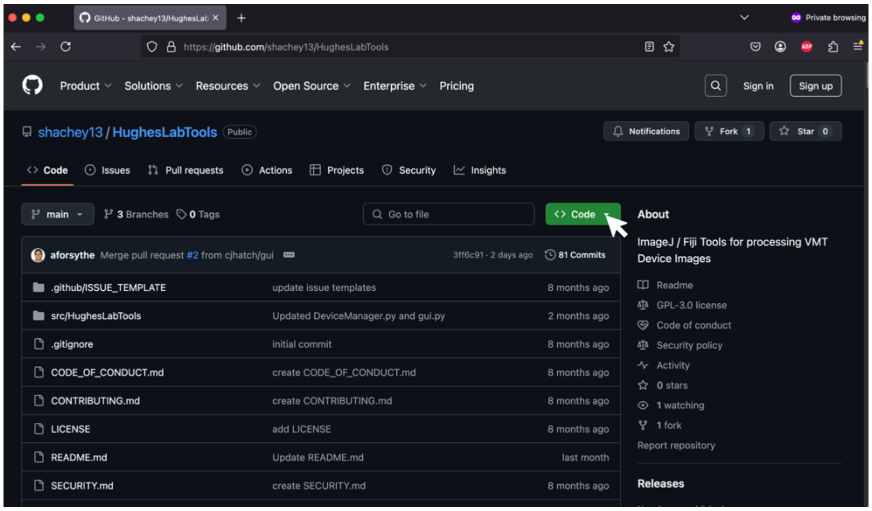

3. Download ZIP

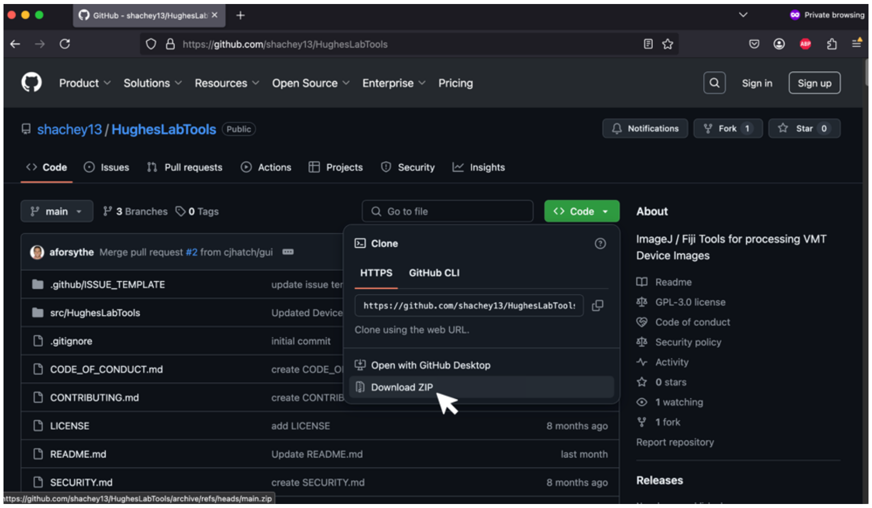

MacOS install

4. Go to the Downloads Folder and unzip the HughesLabTools.zip by clicking on it twice.

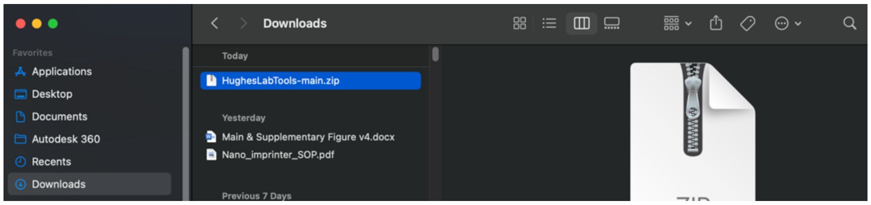

5. Once unzipped, check to ensure the hugheslabtools_install.sh is downloaded. *Note: If you prefer to manually install after this point, follow the WindowsOS commands starting at Step 7*

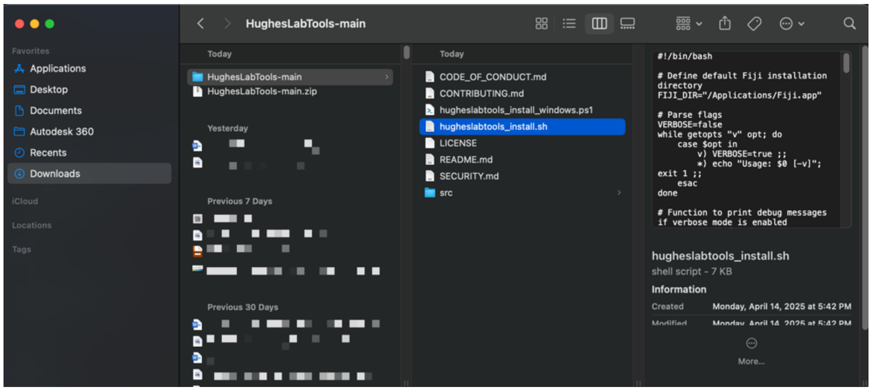

6. Open the Terminal App and change the directory to the Downloads using the command: cd Downloads
7. Ensure the hugheslabtools_install.sh is executable with:
   chmod +x HughesLabTools-main/hugheslabtools_install.sh
8. Run hugheslabtools_install.sh and follow the prompted install instructions using the command:
   HughesLabTools-main/hugheslabtools_install.sh

```
> cd Downloads
> chmod +x HughesLabTools-main/hugheslabtools_install.sh
> HughesLabTools-main/hugheslabtools_install.sh
Fiji is installed in /Applications/Fiji.app. Proceeding with installation...
1) Copy Hughes Lab Tools to Fiji (End-user Mode)
2) SymLink Hughes Lab Tools to Fiji (Developer Mode)
3) Uninstall Hughes Lab Tools
4) Quit
Please choose installation type: 1
Copying Hughes Lab Tools to Fiji ...
Done
>
```

9. Check to make sure the installation was successful

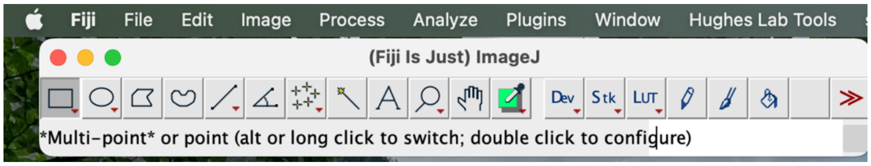

Manual/Windows OS Installation

4. Go to the Downloads folder and right-click the HughesLabTools-main and select Extract files…

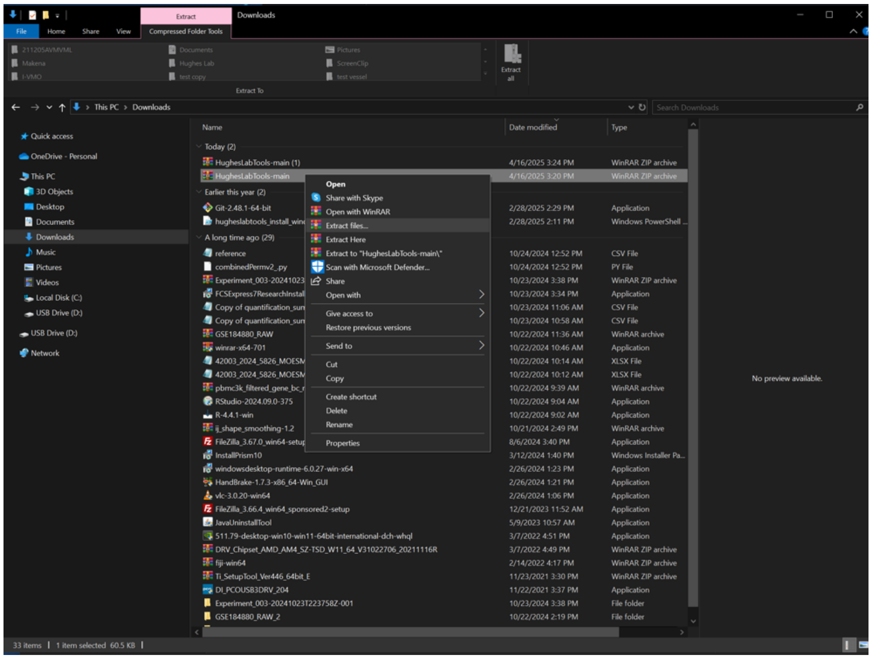

5. Extract the contents of the zipped file by leaving values as the defaults and selecting your location; for this case, it was the Downloads folder

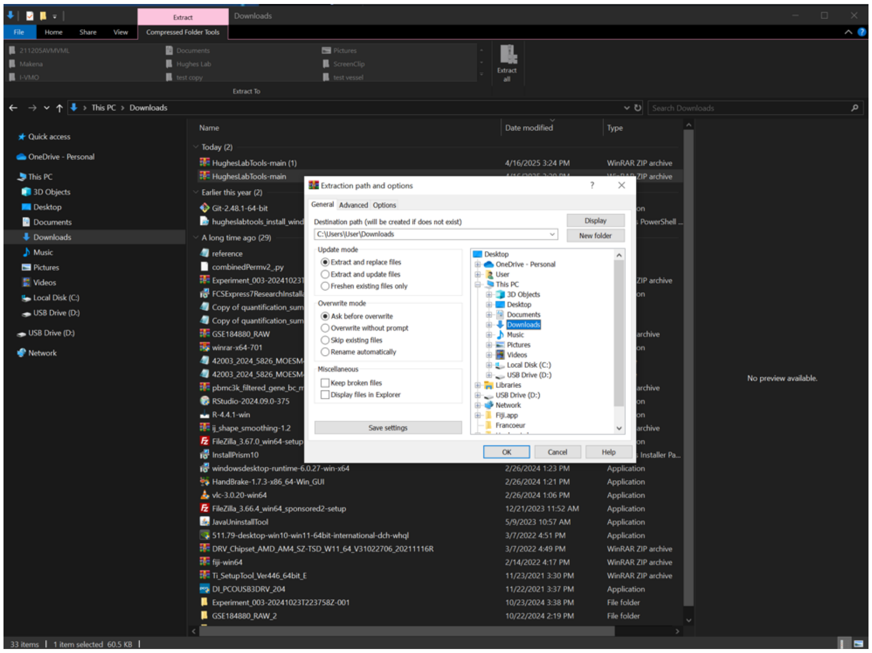

6. Navigate to the FIJI.app installation on your computer and open in a new window to show the contents

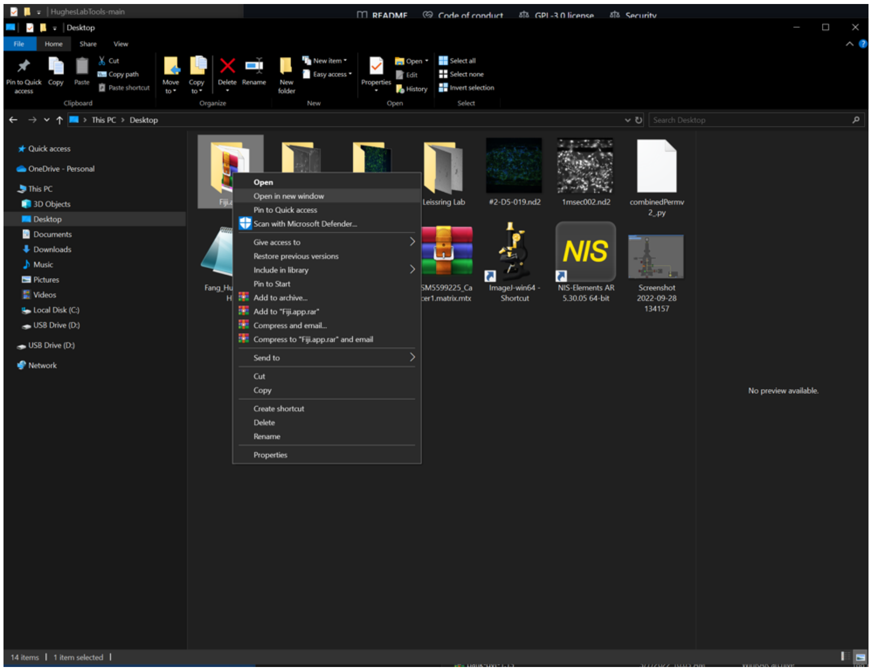

7. In the FIJI/ImageJ directory go to the jars folder and create a new folder called Lib. *Note: For MacOS users right click the FIJI application and select Show Package Contents.*

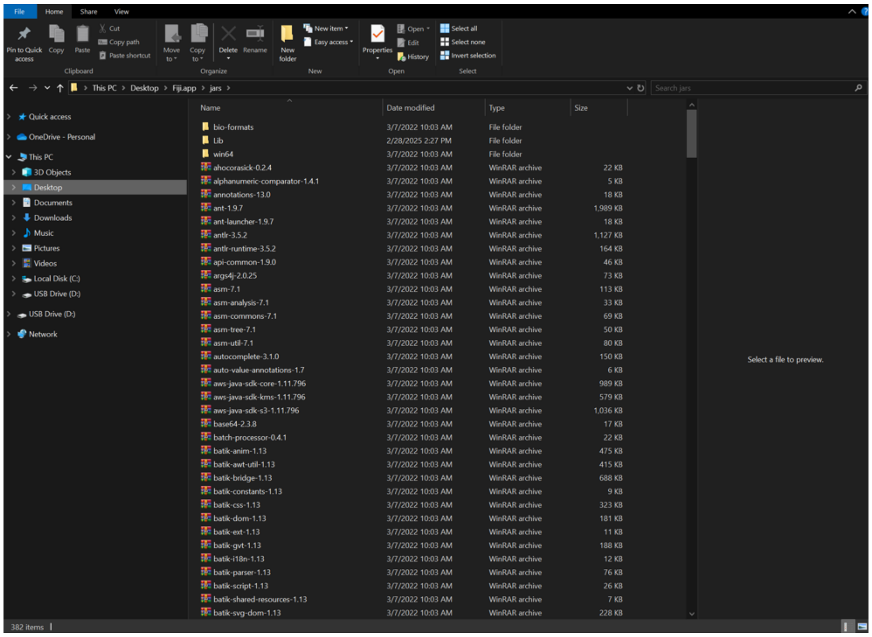

8. Go to HughesLabTools-main > src in the unzipped folder and copy the entire HughesLabTools folder

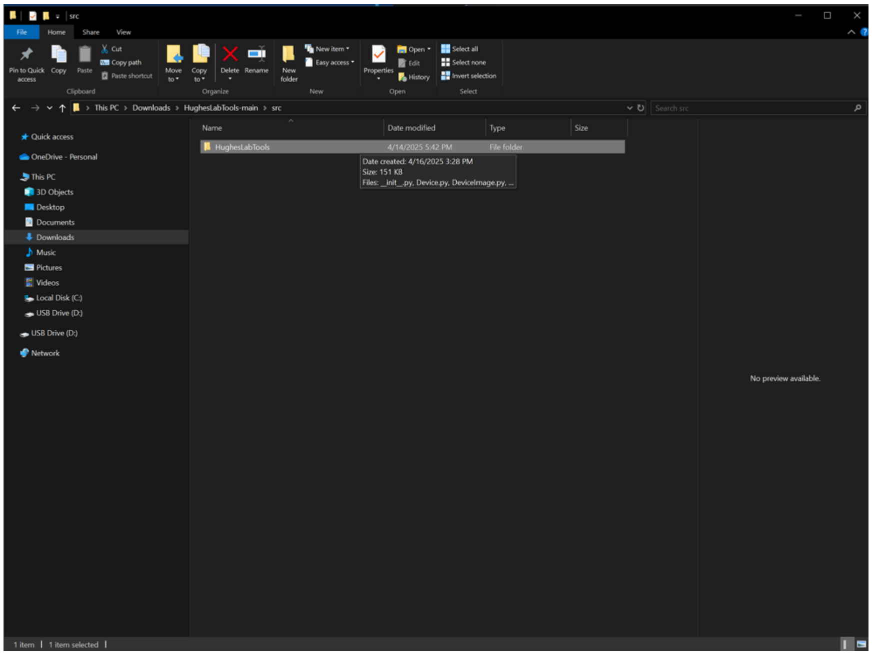

9. Paste the copied folder into the jars > Lib folder
10. Go to the FIJI folder and create a new folder called scripts. Go to the HughesLabTools folder and copy the main_.py and paste it into the scripts folder

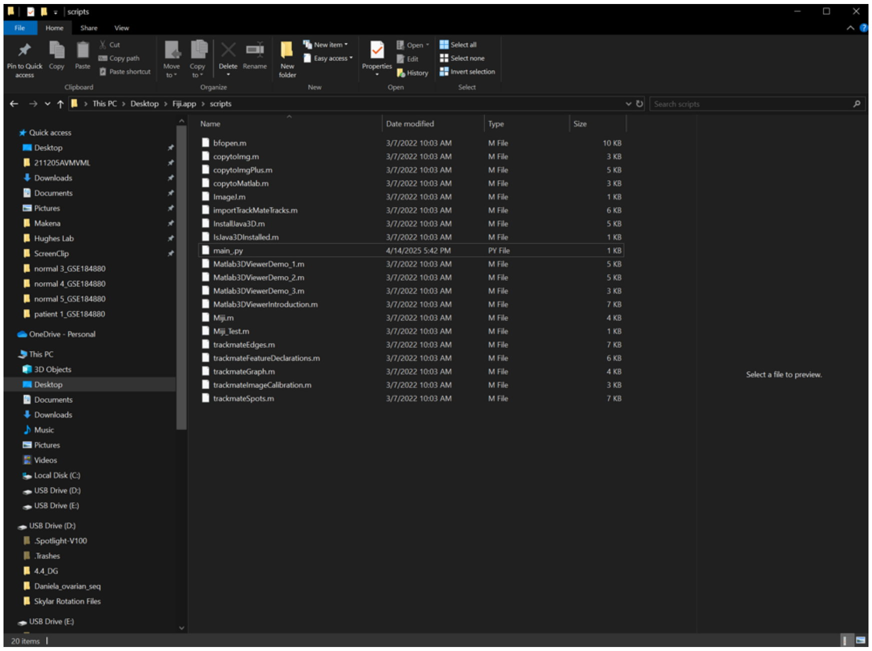

11. Open FIJI/ImageJ and check that the HughesLabTools is now available

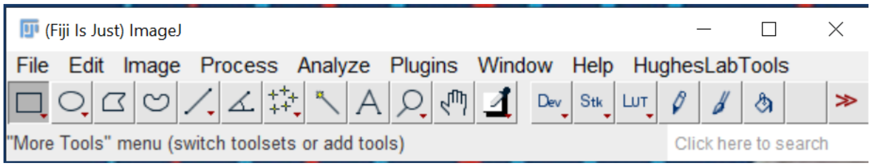

Clone the Git Repository

Ensure Git is installed on your system before proceeding. See https://git-scm.com/ for details.

Open a terminal window and run the following command to clone the repository :

```
git clone git@github.com:shachey13/HughesLabTools.git
```

This will create a folder named 'HughesLabTools' in your current directory.

1.1 MacOS Installation

1. Open the Terminal App.
2. Change into the cloned directory:

```
cd HughesLabTools
```

3. Make the install script executable:

```
chmod +x hugheslabtools_install.sh
```

4. Run the installer:

```
./hugheslabtools_install.sh
```

5. Follow the on-screen prompts to complete the installation.
6. Confirm the installation was successful.

1.2 Manual/Windows OS Installation

7. Open the folder you just cloned (HughesLabTools).
8. Navigate to src, then locate the HughesLabTools folder.
9. Open your FIJI.app installation directory. On Mac, right-click the FIJI application and choose 'Show Package Contents'.
10. Inside the FIJI/ImageJ directory:
    - Go to the jars folder and create a new folder called Lib.
    - Copy the HughesLabTools folder (from src) into the jars/Lib folder.
11. Still inside the FIJI directory, create a new folder called scripts (if it doesn't exist), and also copy the HughesLabTools folder into it.
12. Open FIJI/ImageJ and confirm that the HughesLabTools suite is available
